# A single-cell transcriptomic atlas of the human ciliary body

**DOI:** 10.1007/s00018-022-04559-w

**Published:** 2022-09-26

**Authors:** Bingsheng Lou, Lei Zeng, Xinbo Gao, Xiaobing Qian, Jing Jing Li, Xinyu Gu, Zheng Liu, Keli Liu, Xun Chen, Xiaofeng Lin, Feng Zhang

**Affiliations:** State Key Laboratory of Ophthalmology, Zhongshan Ophthalmic Center, Guangdong Provincial Key Laboratory of Ophthalmology and Visual Science, Sun Yat-Sen University, Guangzhou, 510060 China

**Keywords:** Singe-cell RNA sequencing, Aqueous humor, Glaucoma, Pigmented epithelium, Non-pigmented epithelium, Ciliary muscle, Macrophage, Uveal immunity, Cell–cell communication

## Abstract

The ciliary body critically contributes to the ocular physiology with multiple responsibilities in the production of aqueous humor, vision accommodation and intraocular immunity. Comparatively little work, however, has revealed the single-cell molecular taxonomy of the human ciliary body required for studying these functionalities. In this study, we report a comprehensive atlas of the cellular and molecular components of human ciliary body as well as their interactions using single-cell RNA sequencing (scRNAseq). Cluster analysis of the transcriptome of 14,563 individual ciliary cells from the eyes of 3 human donors identified 14 distinct cell types, including the ciliary epithelium, smooth muscle, vascular endothelial cell, immune cell and other stromal cell populations. Cell-type discriminative gene markers were also revealed. Unique gene expression patterns essential for ciliary epithelium-mediated aqueous humor inflow and ciliary smooth muscle contractility were identified. Importantly, we discovered the transitional states that probably contribute to the transition of ciliary macrophage into retina microglia and verified no lymphatics in the ciliary body. Moreover, the utilization of CellPhoneDB allowed us to systemically infer cell–cell interactions among diverse ciliary cells including those that potentially participate in the pathogenesis of glaucoma and uveitis. Altogether, these new findings provide insights into the regulation of intraocular pressure, accommodation reflex and immune homeostasis under physiological and pathological conditions.

**Supplementary Information:**

The online version contains supplementary material available at 10.1007/s00018-022-04559-w.

## Introduction

The ciliary body is a circular inner ocular structure located behind the iris and in front of the choroid. It consists of the anterior thicker part called *pars plicata*, which is made of many ciliary processes, and the posterior thinner part called *pars plana* [1]. Within the ciliary processes are the epithelium, stromal tissue and blood vasculature. The ciliary processes are attached to the lens by suspensory ligaments, also known as zonular fibers. When the ciliary muscle that locates exteriorly to the ciliary processes contracts, tension on the zonular fibers is relieved and the lens becomes more spherical, thereby enabling focusing on near objects, a process called accommodation reflex [1].

The ciliary epithelium is a bilayer cell alignment comprising the exterior pigmented epithelium (PE) layer and the interior non-pigmented epithelium (NPE) layer. Developmentally, the ciliary epithelium originates from the distal region of the optical cup and is differentiated from two different neuro-epithelial cell origins: NPE and neural retina are derived from the inner layer of the optic cup; and PE and the adjacent retinal pigment epithelium (RPE) are from the outer layer of the optic cup [2, 3]. Previous studies have revealed that the morphogenesis of the ciliary epithelium is controlled by key transcription factors such as PAX6 and OTX1 and a complex signaling network mediated by classic growth factor pathways such as TGFβ, Notch, WNT/β-catenin and Hedgehog [2, 3]. Functionally, NPE constantly generates aqueous humor from the plasma via the transcellular pathway to nourish surrounding avascular tissues, including the corneal endothelium, the lens, and the trabecular meshwork [4]. In contrast, paracellular transport of fluids from blood is strictly restricted due to the blood-aqueous barrier formed by tightly joined intercellular junctions of the ciliary epithelium [5]. The ciliary body (primarily the epithelium) also secrets a variety of neuroendocrine peptides [4] that intimately regulate the circadian rhythms of the aqueous flow and intraocular pressure and extracellular matrix (ECM) proteins [6] that critically contribute to the stabilization and turnover of the inner limiting membrane and the vitreous body.

Like the brain, the eye is one of the few immune privileged sites of the body. Due to the segregated state from the peripheral immune system, the eye contains various myeloid cells that respond to foreign and self-antigens, and locally, tissue-resident macrophages or microglia are the well-characterized immune cell types that mediate baseline immunity in the intraocular tissues [7–9]. Extensive studies from rodent eyes have revealed that the immune cell populations of the healthy ciliary body consist primarily of cells of the myeloid lineage such as monocytes and macrophages; however, comparatively little work has been focused on the immune environment of the human ciliary body [9].

The ciliary body processes contain many fenestrated capillaries to enable quick exchange of oxygen, fluids and soluble factors that are vital to this multifunctional and interactive tissue [8, 10]. However, the ciliary body was previously thought to be physiologically devoid of draining lymphatic vessels. This concept has recently been challenged, partially due to the identification of a functional meningeal lymphatic network that drains the cerebrospinal fluid [11, 12]. Attempts have been made to identify similar phenomena in the eye, but the results were presented without conclusive evidence. In human ciliary body, the presence of D2–40 and LYVE-1 positive lymphatic vessels was reported in one study [13]. This is in contrast to other studies which have shown the absence of LYVE-1 and podoplanin positive lymphatics in human ciliary body with melanoma without extraocular extension, although neo-lymphatic vessels can proliferate into malignant melanomas of the ciliary body with extraocular extension [14, 15].

The high-throughput single-cell RNA sequencing (scRNAseq) technology empowers unprecedented depiction of differential cell populations, functionality-related transcriptomic profile, differentiation status and cell–cell communication at single-cell resolution. A number of scRNAseq studies have been conducted to generate cell atlas of various human ocular tissues including the retina, choroid, limbal epithelium and aqueous humor outflow tissues [16–22]. In this study, we performed comprehensive single-cell transcriptional profiling of the ciliary body from human donor eyes. Unique gene expression patterns of epithelium, smooth muscle cell (SMC), endothelial cell (EC), immune cell and other stromal cell populations within the ciliary body were identified. We also placed emphasis on the essential molecular elements involved in the control of aqueous humor inflow, blood-aqueous barrier and physiological interactions between diverse ciliary cells. Taken together, our study provides a detailed molecular and cellular portrait of human ciliary body that has wide implications for the understanding of the cellular basis of intraocular fluid homeostasis and immune environment.

## Methods

### Human donor eyes

ScRNAseq was performed on eyes of 3 human donors obtained from Guangdong Eye Bank, Zhongshan Ophthalmic Center, Sun Yat-sen University. The eye pair 1 was from a 41-year-old male donor patient who died due to cerebral hemorrhage. Pair 2 was from a 33-year-old male donor who died from brainstem hemorrhage. Pair 3 was from a 55-year-old male donor whose cause of death was lung cancer. All donors were not diagnosed with ocular diseases. Characteristics of the donors are reported in Suppl. Table 1. Acquisition of the donor eyes for scRNAseq and immunohistological studies were approved by the Medical Ethics Committee of Zhongshan Ophthalmic Center, Sun Yat-sen University, and performed in agreement with the tenets of the Declaration of Helsinki.

### Tissue dissection

The eyes after corneal excision were immediately immersed in PBS containing 50 U/ml penicillin–streptomycin on ice (please refer to Suppl. Table 1 for detailed information of the elapsed time for the generation of live single cell suspension and of cDNA sequencing libraries after the donor's death) and bisected along the equator so that the anterior and posterior halves were separated. The lens and surrounding zonular fibers were then removed from the anterior half of the eye, followed by cutting the shell of the anterior half into four radial wedges. A microsurgical scissor was used to carefully cut through each wedge along the ora serrata that separates the retina and ciliary body. After removal of the iris and sclera from each wedge, the ciliary body tissue was collected and then subjected to cell dissociation.

### Cell dissociation and viability test

The ciliary body tissues from each pair of the eyes were incubated in 25 ml PBS containing 0.04% BSA, 1.2 U/mL dispase and 50 U/mL penicillin–streptomycin at room temperature for 15 min. Thereafter, trypsin (final conc. 1.33 mg/mL), hyaluronidase (final conc. 0.67 mg/mL), kynurenic acid (final conc. 0.2 mg/mL), MgCl_2_ (final conc. 3.2 mM) and CaCl_2_ (final conc. 0.1 mM) were added in the digestion buffer, followed by another incubation of the mixture at 37 °C for 15 min with occasional shaking. The digestion was terminated by the addition of heat-inactivated fetal bovine serum (FBS, final conc. 10%) and the tissues were further dissociated by repeated pipetting and gentle mincing and filtered through a 70-μm strainer and then a 40-μm strainer. Cells were rinsed with 0.04% BSA/PBS for 3 times after centrifugation at 1,500 rpm for 5 min and re-suspended with 3 ml 0.04% BSA/PBS.

For cell count and viability test, cells were dispersed by gentle pipetting and passed through a 35-μm strainer. A small aliquot of the cell suspension was then mixed with an equal volume of Acridine Orange/Propidium Iodide solution for staining. Cell number and viability were measured by the Countstar automated cell analyzer. Approximately 400,000–800,000 ciliary cells with > 80% viability were obtained from each preparation.

### Single-cell RNA sequencing

A BD Rhapsody analysis system was used to prepare single-cell whole transcriptome. Cell suspension was loaded into the BD Rhapsody cartridge containing a microwell array with > 220,000 partitions. Single cell capture was achieved by random distribution of single cells that settled into the microwells by gravity, followed by the addition of beads with oligonucleotide barcodes for pairing with the cells. After cell lysing, the barcoded beads containing the captured mRNAs were retrieved, washed and then subjected to reverse transcription and Exonuclease I digestion. Single-cell transcriptomic sequencing libraries were prepared by following a series of PCR steps including random priming and extension (RPE), RPE PCR and whole-transcriptome amplification (WTA) index PCR according to the manufacture’s instruction. Normalized libraries were sequenced on NovaSeq Illumina with a 150 bp paired-end run.

### Processing of scRNAseq data

Quality control and preprocessing of the FASTQ sequencing data was performed using the *fastp* preprocessor tool with default parameters [23]. The UMI-Tools method [24] that models the errors in the UMI sequence was used to improve quantification accuracy. The UMI-based clean reads were then mapped to the human genome (Ensemble version 100), followed by integration of the datasets from the three sequencing samples into one large matrix dataset using the downsampleReads function in the DropletUtils package [25]. A Seurat package (Version 4.1.0) was used for further processing of the matrix data that contained expression values of a total of 20,590 individual ciliary cells before filtering. After rare reads/genes (found in < 10 cells), low-quality cells (with < 250 detected genes or > 40% reads mapping to the mitochondrial genome) and presumed doublets (DoubletScore > 35) identified using the scDblFinder (v1.6.0) package [26] were filtered out, a total of 14,563 cells from the three human donors were retained. The Seurat SCTransform function was then applied to normalize the clean data and regress unwanted variation based on total read counts, mitochondria gene rate and the top 4,000 high variable genes in each cell. The final gene expression matrix (UMI counts per gene per cell) was then obtained. Please refer to Suppl. Table 2 for key metrics of the human ciliary scRNAseq dataset before and after the filtering steps.

### Cell clustering analyses

Cell clusters were acquired via the graph-based clustering approach in Seurat. The FindNeighbors input dimensions of reduction was set to 40 which was determined by principal component analysis (PCA) via the RunPCA function, and the Findclusters resolution parameter was set to 0.1. The projected clusters were visualized after nonlinear dimensional reduction by Uniform Manifold Approximation and Projection (UMAP) and annotated using known marker genes. A top 50 ranked list of predicted markers (differentially expressed genes, refer to Suppl. Table 3) for each cell cluster were also identified by the FindAllMarkers function according to the following threshold metrics: Log2FoldChange > 0.25; one-sided Wilcoxon rank sum test *p* < 0.01; and pct.1 > 10%. Alternatively, to identify potential marker genes of both PE and NPE populations, the FindMarkers function was employed via comparing each PE/NPE population with the group of all other non-epithelial ciliary cells using the following threshold metrics: Log2FoldChange > 0.25; one-sided Wilcoxon rank sum test *p* < 0.05; pct.1 (in PE/NPE) > 25%; and pct.2 (in all other ciliary cells) < 5%), and the resulted 40 common genes for both PE and NPE was obtained (refer to Suppl. Table 4).

Subclusters in the SMC, endothelial cell and macrophage populations were determined through the aforementioned normalization, regression and graph-based clustering pipeline with differential PCA and resolution parameters and annotated based on expression pattern of previously published gene markers (refer to Suppl. Table 6 for gene abbreviation list) as visualized on UMAP or examined by immunofluorescent staining.

Additionally, monocytes and macrophages in human ciliary body as well as the microglial cell clusters from human retinas, which are also resident mononuclear phagocytes, were subjected to clustering analysis. We obtained three sets of raw counts data of the microglial cell clusters from existing retinal scRNAseq datasets [20–22] and combined with our monocyte/macrophage raw counts data. The four datasets were normalized using the SCRAN computeSumFactor function with the default parameters followed by batch effect correction via the mnnCorrect function as described previously [27]. Clusters of these myeloid cells were then identified through the graph-based clustering pipeline, annotated and visualized on UMAP.

Finally, the FindMarkers function was implemented to obtain differentially expressed genes (DEGs; fold change > 1.5; one-sided Wilcoxon rank sum test *p* < 0.05; and pct > 10%) between two relevant clusters/subclusters. KEGG functional enrichment analysis was performed on the DEGs using a hyper-geometrical statistical test with *p* < 0.05 considered statistically significant. Gene set enrichment analysis (GSEA) was also employed using the indicated GO pathways (Suppl. Table 7).

### Cell–cell communication analysis

To infer cell–cell communication networks among human ciliary cells, we implemented the publicly available CellPhoneDB (v.2.0) tool which incorporates a repository of 501 secreted proteins (ligands) and 585 membrane proteins (receptors) [28]. These proteins are involved in 1396 ligand-receptor interactions from chemokines, growth factors/hormone, ECMs and membrane proteins such as receptor tyrosine kinase (RTK) families and integrins with the accurate subunit architecture of protein complex integrated. Using the normalized count data of the ciliary single-cell transcriptomics, ligand–receptor interaction scores were computed between two cell types according to the expression levels of the ligands and receptors and the percentage of cells expressing these proteins. A one-sided Wilcoxon rank-sum test was performed on the interaction scores, with *p* < 0.05 considered statistically significant. The predicted cell–cell communication network comprising complex protein interactions between specific cell populations was then generated.

### Mice

To visualize Prox1 + lymphatic vessels in vivo, we generated *Tg-Prox1-EGFP* transgenic mice (Gem Pharmatech Co. Ltd, China). An EGFP-BGHPolyA-FRT expression cassette was introduced by recombineering at the start codon (ATG) of Prox1 gene in bacterial artificial chromosome (BAC) clone RP23-385H16 that contains an approximately 215 kb mouse Prox1 genomic DNA (including the entire Prox1 gene and the 134 kb sequence upstream of the start codon and 41.9 kb sequence downstream of the stop codon). Following microinjection of the transgenic construct into mouse donor zygotes, the resulting founder *Tg-Prox1-EGFP* BAC mice were propagated on a C57BL/6J background. Genotyping was performed by detecting the Prox1-EGFP transgene using the following primer pair:

5′-CCATCTTAAACCAGTGGAGCAGA-3′

5′-AACTTGTGGCCGTTTACGTCG-3′

Faithful lymphatic expression of the Prox1-EGFP transgene was confirmed in LYVE1 stained limbus lymphatic vessels and other lymphatic vascular beds.

The Institutional Animal Care and Use Committee (IACUC) of Zhongshan Ophthalmic Center, Sun Yat-sen University has approved the mouse studies. All animal experiments were in accordance with Use of Animals in Ophthalmology and Vision Research.

### Immunofluorescence staining

Human donor eyes were fixed in 4% paraformaldehyde (PFA)/PBS overnight, infiltrated with 30% sucrose for 48 h, embedded in OCT medium and processed for cryosectioning and standard histological examinations. Cross-Sects. (10 μm in thickness) of the eye were blocked with PBS containing 0.1% Triton X-100 and 5% donkey serum for 1 h and incubated with primary antibodies at 4 °C overnight and fluorescently labeled secondary antibodies and DAPI at room temperature for 45 min, with adequate PBS washes after each incubation. After mounting, the sections were imaged under a Nikon CS2 confocal microscope.

For tissue whole mount staining, eyes were harvested from postnatal day 21 (P21) *Tg-Prox1-EGFP* transgenic mice and fixed in 4% PFA/PBS overnight. The limbus and ciliary body tissues were dissected from the fixed eyes under a stereomicroscope, followed by blocking in a blocking buffer (0.5% triton X-100, 5% donkey serum, 1% BSA in PBS) and incubations with anti-LYVE1 antibodies (diluted in blocking buffer) for two days at 4 °C and appropriate secondary antibodies at 4 °C overnight. After washing, the immune stained tissues were mounted using VECTASHIELD mounting medium containing DAPI and photographed with a Nikon CS2 confocal microscope.

## Results

### Identification of major cell types in human ciliary body using scRNAseq

To determine the precise cell constitution in the human ciliary body, we performed scRNAseq experiments on the ciliary body tissue from 3 pairs of human donor eyes, with each eye pair used as a sequencing sample (Suppl. Table 1). The pieces of ciliary body tissue including the *pars plicata* and *pars plana* regions (Suppl. Fig. 1) without adjacent ocular tissues were carefully dissected and dispersed into single cell suspension as described in Methods. Individual cells were captured using the BD Rhapsody platform and then subjected to scRNAseq analysis. A total of 20,590 individual ciliary cells from the three scRNAseq experiments were initially obtained and integrated into a single matrix dataset (Suppl. Table 2). After filtering out rare reads, low-quality cells and presumed doublets (see Methods), single-cell transcriptome of a total of 14,563 individual ciliary cells were retained, with each sequencing sample ranging from 3848 to 5370 cells (Suppl. Table 2). The average genes per cell was 1778 and the total gene number reached 16,088, in which 15,852 genes were common genes detected in all three samples (Suppl. Table 2 and Suppl. Fig. 2A).

**Fig. 1:**
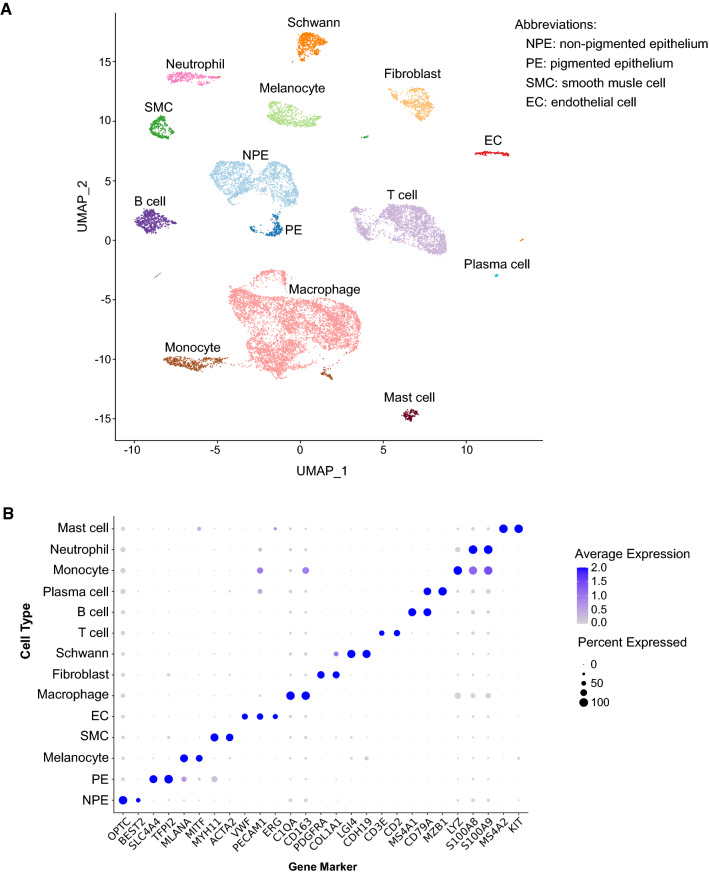
Identification of cell types in human ciliary body using scRNAseq. **A** Uniform manifold approximation and projection (UMAP) visualization of 14 cell types identified from a total of 14,563 ciliary single cell transcriptome in 3 pairs of human donor eyes. Cell clusters are colored and labelled with cell-type annotation. **B** Dot plot of gene markers used to annotate the 14 ciliary cell types in A. Dot size represents frequency of cells in the indicated clusters that express a particular gene marker. Color scale shows the average of log-transformed read counts of each gene

We then sorted the cells into 14 individual clusters, as visualized by UMAP, based on their gene expression profiles and annotated the clusters using known marker genes (Fig. 1A, B and Suppl. Table 3). The cell types are NPE, PE, SMC and various cell populations that reside in the ciliary process stroma including EC, fibroblast, melanocyte, Schwann cell and immune cell (Fig. 1A, B, Suppl. Fig. 2B). In agreement with previous immunohistological studies [9], we found that myeloid cells including monocytes, macrophages, neutrophils and mast cells contributed to the majority of the immune cell population in the ciliary body (Fig. 1A, B, Suppl. Fig. 2B). Lymphocytes including T cells, B cells and plasma cells, which were most likely from the blood in the human donor eyes, were also identified (Fig. 1A, B, Suppl. Fig. 2B). These cell types were found in all three pairs of human eyes, although the proportions of different types varied across the three eye pairs (Suppl. Fig. 2C). In particular, eye pair 3 was found to have relatively increased fraction of macrophages but decreased populations of T cells, B cells, monocytes, and neutrophils compared to the other two pairs. Pairs 1 and 2 also had differential fractions of the cell types other than NPE, macrophages, T cells and mast cells (Suppl. Fig. 2C).

### Analysis of gene expression patterns involved in specialized functionality of the ciliary epithelium

**Fig. 2:**
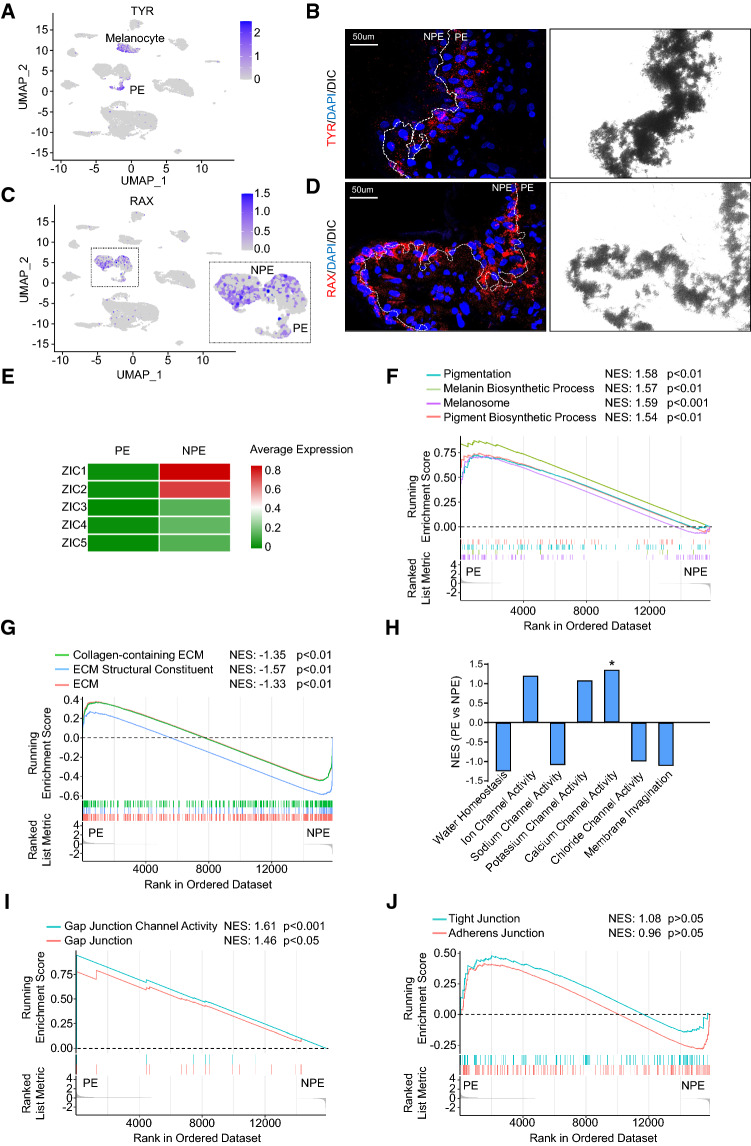
Comparison of gene expression profile between ciliary PE and NPE. **A** UMAP plot of TYR expression in each ciliary cell. The logged expression level of each gene is labelled by color intensity scale. **B** Immunofluorescent staining of TYR (red) and DAPI (blue) and the corresponding DIC phase-contrast images of the sectioned human ciliary process. Dot line highlights the boundary of ciliary NPE and PE layers based on the distribution of pigmentation granules. Note enriched TYR expression in PE area. **C** UMAP plot of RAX expression in each ciliary cell. The logged expression level of each gene is labelled by color intensity scale. **D** Immunofluorescent staining of RAX (red) and DAPI (blue) and the corresponding phase-contrast images of the sectioned human ciliary process. Dot line highlights the boundary of ciliary NPE and PE layers based on the distribution of pigmentation granules. Note expression of RAX in both NPE and PE layers. **E** Heatmap showing averaged expression levels of Zic family members in ciliary PE and NPE populations. Gene set enrichment analysis (GSEA) showing expression profile of melanin synthesis (**F**) and ECM constituent and organization (**G**) pathways in ciliary PE and NPE populations. NES Normalized enrichment score. *ECM* extracellular matrix. **H** Normalized enrichment scores of water hemostasis, ion channel and plasma membrane invagination pathways determined by GSEA in ciliary PE vs NPE populations. *NES* Normalized enrichment score. **I**, **J** GSEA showing expression profile of the indicated cell junction pathways in ciliary PE and NPE populations. *NES* normalized enrichment score

The ciliary PE and NPE appear structurally and functionally different. Indeed, scRNAseq revealed two distinct epithelial cell clusters representing PE (SLC4A4+/TFPI2+/MLANA+ [29]) and NPE (OPTC+/Best2+ [30, 31]), respectively (Fig. 1A, B). Of note, melanocyte, which also expressed MLANA, was annotated due to its specific expression of MITF [32] (Fig. 1B). Immunofluorescence staining demonstrated that ciliary PE cells lining the outer layer of the ciliary processes with the characteristic brownish black appearance and melanocytes located in the stromal area have strongly enriched expression of tyrosinase (TYR) (Fig. 2A, B), a copper-containing enzyme that catalyzes the formation of melanin from tyrosine [33]. Furthermore, we identified that the retinal and anterior neural fold homeobox (RAX) gene is specifically expressed in the NPE and PE clusters (Fig. 2C, D, Suppl. Fig. 3A and Suppl. Table 4), demonstrating a new marker for distinguishing the ciliary epithelium from other ciliary cells. The genes specifically expressed in both PE and NPE also include other homeobox genes, such as PAX6, SIX3 and LHX2 [34–37], common markers for the epithelium, such as DSP (encoding desmoplakin), DSG2 (encoding desmoglein 2) and EGFR, and ion/water channel genes, etc. (Suppl. Fig. 3A and Suppl. Table 4).

Next, we compared PE and NPE transcriptome and found only a small proportion of genes (260 out of 16,088 genes) were differentially expressed (fold change > 1.5; *p* < 0.05) between these two cell types (Suppl. Fig. 3B and Suppl. Table 5), indicating similar transcriptomic features of the ciliary epithelium bilayer. In agreement with a previous study [38] that compared the single-cell transcriptome between mouse PE and NPE, we also found that human NPE expressed higher levels of the ZIC family transcription factors ZIC1 and ZIC2 than PE (Fig. 2E). Beyond this, gene set enrichment analysis (GSEA) revealed that PE unsurprisingly expressed high levels of genes related to melanin/pigment biosynthesis (Fig. 2F), whereas NPE has significantly enriched expression of ECM components such as collagen (Fig. 2G), supporting a preferential role of NPE in the turnover/maintenance of basement membrane of the ciliary epithelium. With respect to the structural constituents required for aqueous humor secretion, we confirmed that PE and NPE expressed similar levels of genes responsible for water homeostasis and ion exchangers, except for those genes that are involved in the control of calcium channel activity which were slightly enriched in PE (Fig. 2H). The two types of epithelial cells also possessed comparable levels of plasma membrane invagination regulators (Fig. 2H), reflecting the comparable demands of fluid transmembrane dynamics by the ciliary epithelium bilayer. Interestingly, we found that the gene sets responsible for gap junction (i.e., connexins) assembly and channel activity were significantly enriched in PE over NPE (Fig. 2I), indicating that the PE layer might undertake more gap junction-mediated fluid transmission than the NPE layer. In addition, PE and NPE expressed similar levels of tight junction complexes (Fig. 2J) that are indispensable for the formation and maintenance of the blood–aqueous barrier [5].

### Lack of lymphatics in mammalian ciliary body

**Fig. 3:**
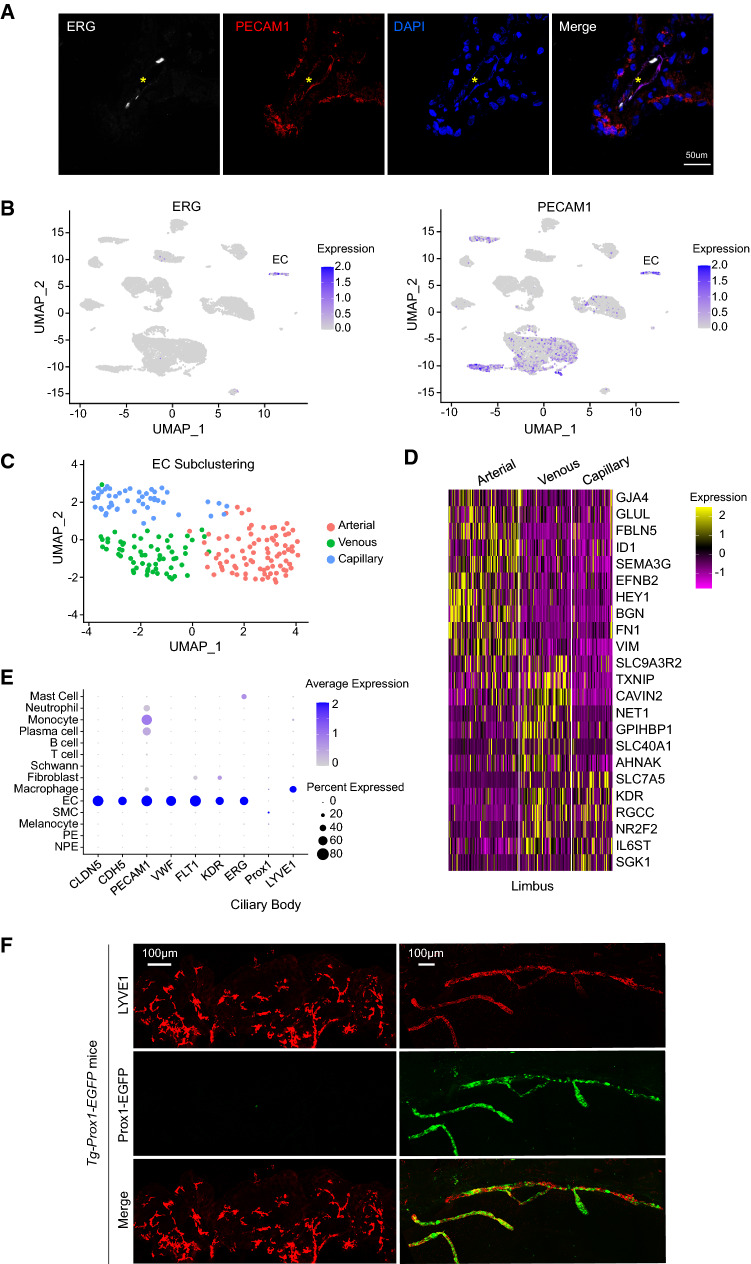
Characterization of expression of blood and lymphatic endothelial cell genes in human ciliary body. **A** Immunofluorescent staining of PECAM1 (red), ERG (white) and DAPI (blue) of cross-sectioned human ciliary body. *Note the PECAM1 and ERG labelled blood vessel in the stromal area of the ciliary process. **B** UMAP plot of logged expression levels of ERG and PECAM1 in ciliary cell clusters. **C** UMAP visualization of arterial, venous and capillary EC subclusters in human ciliary body. *EC* endothelial cell. **D** Heatmap showing expression profile of specific EC markers in the subclusters in **C**. **E** Dot plot of expression levels and frequency of the indicated blood and lymphatic EC markers in human ciliary cells. **F** Whole-mount immunostaining of LYVE1 (red) and Prox1-EGFP (green) in P21*Tg-Prox1-EGFP* mouse ciliary body and limbus areas. Note that LVVE1+/Prox1-EGFP+lymphatic vessels are present in the limbus area but not in the ciliary body

The ciliary body is replenished by a complex network of blood vessels, but whether it contains lymphatics is under debate. Using antibodies against endothelial cell markers PECAM1 and ERG, we were able to label the endothelial cell population in the center of the ciliary process region (Fig. 3A, B). Following subclustering analysis of the endothelial cells, we could identify the classic blood endothelial cell divisions including venous, capillary and arterial ECs which differentially expressed various vascular EC markers (Fig. 3C, D). However, we noted that the expression of lymphatic endothelial cell marker gene Prox1 and LYVE1 were completely absent in the ciliary endothelial cell population that expressed canonical EC genes, such as CLDN5, CDH5, PECAM1, VWF, FLT1, KDR and ERG (Fig. 3E). Immunofluorescent staining of LYVE1 in a *Tg-Prox1-EGFP* transgenic mouse model confirmed that the ciliary body lacked mature lymphatic vessels, although many sparse cells were labelled as LYVE1 positive (Fig. 3F). This is in stark contrast to the limbus region where abundant Prox1-EGFP+/LYVE1+ lymphatic vessels were present (Fig. 3F). Taken together, our data demonstrated that there are no classic lymphatics in mammalian ciliary body at physiological baseline condition.

### Comparison of nonvascular ciliary muscle and vascular smooth muscle cells

**Fig. 4:**
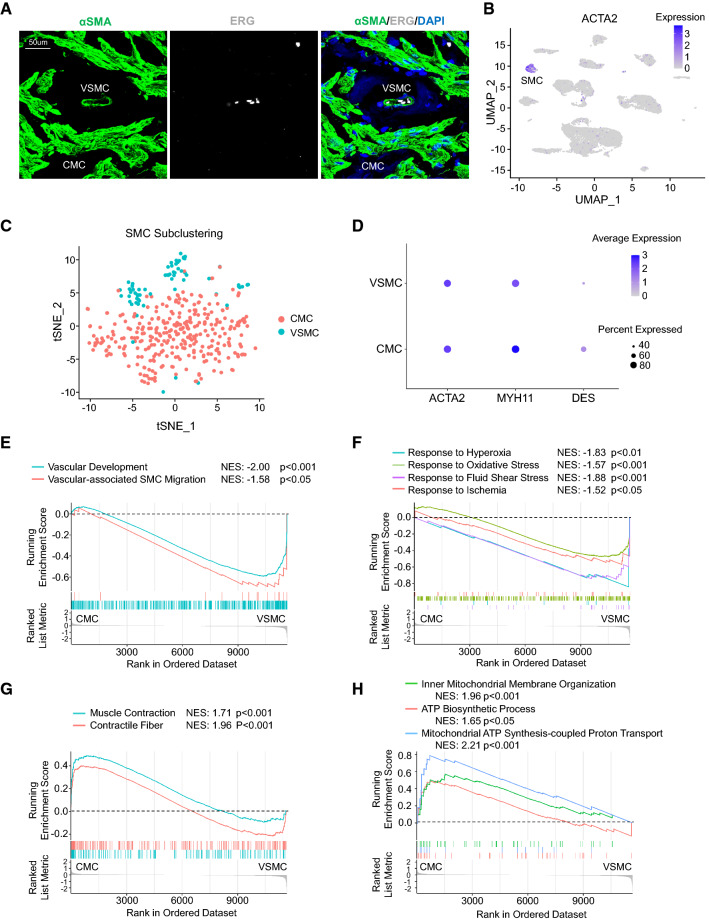
Identification of nonvascular ciliary muscle cell and vascular smooth muscle cell (VSMC) in human ciliary body. **A** Immunofluorescent staining of αSMA, ERG and DAPI (blue) of cross-sectioned human ciliary body. *CMC* ciliary muscle cell. *VSMC* vascular smooth muscle cell. **B** UMAP plot of logged expression levels of ACTA2 (α smooth muscle actin, αSMA) in ciliary cell clusters. **C** tSNE visualization of CMC and VSMC subclusters. *SMC* smooth muscle cell. *CMC* ciliary muscle cell. *VSMC* vascular smooth muscle cell. *tSNE* T-distributed stochastic neighbourhood embedding. **D** Dot plot of expression levels and frequency of SMC markers in CMC and VSMC subclusters in **C**. GSEA showing expression profiles of vascular development (**E**), stress responses (**F**), contractile fiber formation (**G**), and mitochondrial ATP biosynthesis and proton transport (**H**)-related pathways in CMC and VSMC subclusters. *NES* Normalized enrichment score

The ciliary smooth muscle is one of the key elements of the eye that provides intrinsic contraction force during accommodation. Immunofluorescent staining of α smooth muscle actin (αSMA) identified two populations of smooth muscle cells in human ciliary body: one represents vascular smooth muscle cell (VSMC) that covers ERG + blood ECs; the other one is nonvascular ciliary muscle cell (CMC) (Fig. 4A, B). With the scRNAseq subclustering analysis, we confirmed that two SMC subclusters with specific expression of the canonical smooth muscle cell markers ACTA2 (encoding αSMA) and MYH11 were present (Fig. 4C, D). The larger subcluster was assigned as nonvascular CMC due to its enriched expression of desmin, whereas the smaller sub-cluster was annotated as VSMC [39, 40] (Fig. 4C, D). GSEA was able to confirm significantly enriched pathways responsible for vascular development and vascular associated SMC migration in the VSMC subpopulation compared to the CMC subpopulation (Fig. 4E). Consistent with its roles in vascular functioning, VSMC also had overall higher expression of gene sets required for stress responses, such as fluid shear, hyperoxia, ischemia and oxidative stress (Fig. 4F). In contrast, the CMC subpopulation exhibited higher expression of genes for muscular contractile fiber formation (Fig. 4G), supporting the main role of ciliary muscle contraction in accommodation of the lens. Finally, significantly enriched expression of genes responsible for mitochondrial ATP biosynthesis and proton transport was also noted in CMC, but not in VSMC (Fig. 4H), indicating the extraordinarily high demand for ATP as an instant source of energy in this type of muscle.

### Transitional states of the ciliary macrophage

**Fig. 5:**
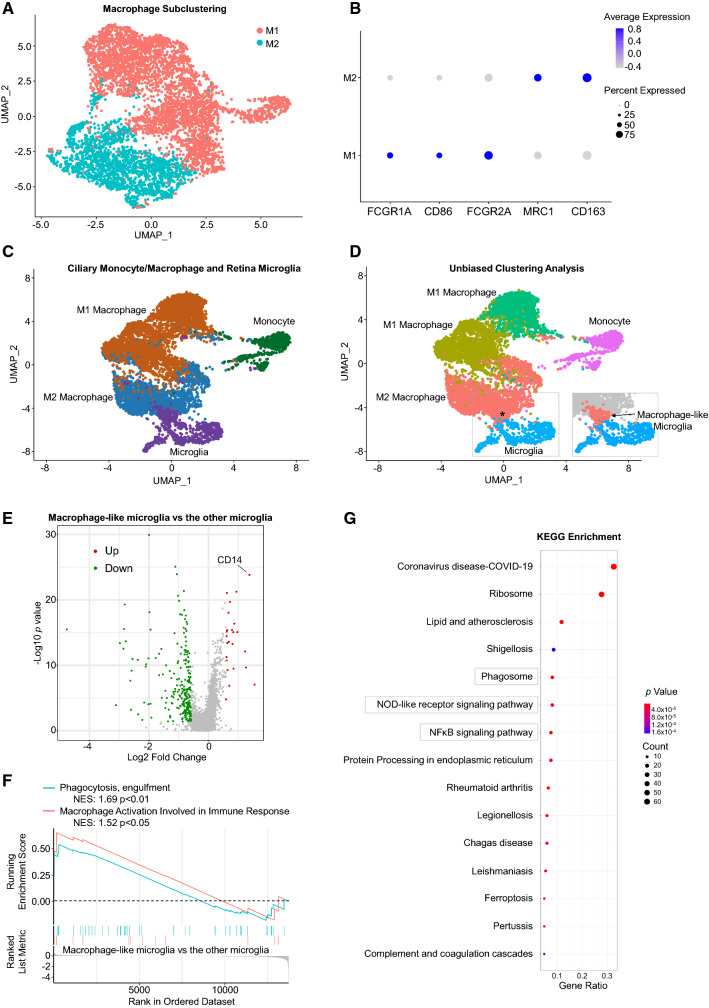
Transitional states of the ciliary macrophage. **A** UMAP visualization of M1- and M2-polarized subclusters of macrophages in scRNAseq from human ciliary body. **B** Dot plot of expression levels and frequency of selected macrophage activation markers in the macrophage subclusters in **A**. **C** UMAP visualization of human ciliary monocyte/macrophage and human retinal microglia. **D** UMAP plot of unbiased clustering of the cells in **C**. *Note that a small proportion of retinal microglia is populated with ciliary M2-polarized macrophages and annotated as macrophage-like microglia. **E** Volcano plot analysis of DEGs between macrophage-like microglia and the other microglia subpopulations. Upregulated and downregulated genes are labelled in red and green, respectively. Note that CD14 is significantly upregulated in the macrophage-like microglia subpopulation. **F** GSEA showing expression profiles of phagocytosis and macrophage activation pathways in the indicated retina microglia subpopulations. NES: normalized enrichment score. **G** KEGG enrichment analysis of the DEGs in **D**. Shown are top 15 enriched pathways. Dot size represents number of DEGs in the indicated pathways. *p* values are indicated by color intensity

Macrophage was one of the greatest contributors to the cell constituents in our scRNAseq analysis. Subclustering analysis on the entire macrophage population was able to segregate this cell population into two transcriptionally distinct subclusters (Fig. 5A). Analysis of the expression of macrophage surface markers and transcription factors revealed that cells in the relatively larger macrophage subcluster had enriched expression of MHC class II genes, FCGR1A, CD86 and FCGR2A (Fig. 5B), consistent with a pro-inflammatory (M1) phenotype [41]. The relatively smaller subcluster was annotated as anti-inflammatory (M2) macrophage [41] due to higher expression of CD163 and MRC1 genes (Fig. 5B). Interestingly, the subclustering analysis of the macrophage population from each individual sequencing sample indicated that most macrophages in the eye pairs 1 and 2 were polarized to M1 type whereas the eye pair 3 had a higher fraction of M2 macrophages (Suppl. Fig. 4). This reflects the heterogenicity of M1/M2 polarization in the ciliary macrophage populations among different human donors, possibly due to their individual healthy conditions and age.

Next, we combined our monocyte/macrophage data with the raw counts of microglia clusters from existing human retinal scRNAseq datasets [20–22] to profile their transcriptional signatures. Unbiased clustering analysis identified 5 segregated subpopulations from the merged cell data, corresponding to monocyte, microglia, M2 macrophage and subclusters of M1 macrophage (Fig. 5C, D). This demonstrates the diversity and heterogenicity of cells of myeloid lineage in the ciliary body and retina. Intriguingly, we noted that a small proportion of microglia were populated with most M2 macrophages in the UMAP plot (Fig. 5D), reflecting their macrophage-like transcriptomic feature. These macrophage-like microglia possessed significantly higher expression levels of the monocyte/macrophage cell marker CD14 [42] (Fig. 5E) and were enriched in pathways responsible for macrophage activation and phagocytosis (Fig. 5F). Therefore, this microglia subpopulation was proposed to be derived from the ciliary M2 macrophage population. This is in line with the observation that the ciliary macrophage serves as one of the origins of the repopulated microglia in the mouse retina after microglia depletion [43]. Gene expression profiling analysis of the microglia subpopulations further revealed that macrophage-like microglia differentially expressed genes involved in phagosome formation and certain immunogenic signaling pathways such as NFκB and NOD-like receptor pathways (Fig. 5G), indicating that these signals might play a role in regulating microglia/macrophage transitional states.

### Cell–cell communications in human ciliary body

**Fig. 6:**
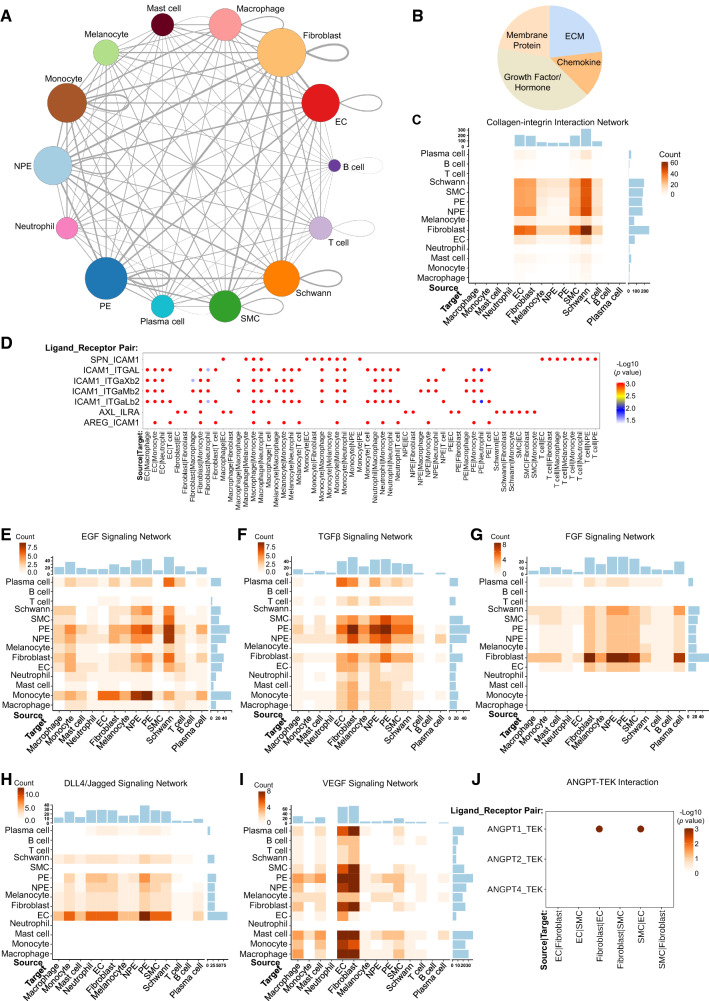
Identification of potential cellular interactions in human ciliary body. **A** Circo plot showing predicted cell–cell communication among all 14 human ciliary cell types by CellPhoneDB. The weights of node and cord size represent the number of significant ligand-receptor pairs between two cell types. **B** Pie chart of the 4 categorized cell–cell interactions in human ciliary cells. Note that cell–cell interactions were categorized into secreted protein (chemokine, ECM and growth factor/hormone)- and membrane protein-mediated interaction according to the nature of the ligands. ECM extracellular matrix. **C** Heatmap showing proportion of significant collagen-integrin interactions among the 14 human ciliary cell types. Color scale represents number of significant interactions. **D** Dot plot overview of all significant cell–cell interactions containing IL15RA and ICAM1 as either ligand or receptor. *p* values are indicated by color intensity. Heatmap showing proportion of significant EGF (**E**), TGFβ (**F**), FGF (**G**), DLL4/Jagged (**H**) and VEGF (**I**)-mediated interactions among the 14 human ciliary cell types. Color scale represents number of significant interactions. **J** Dot plot of significant cell–cell interactions mediated by ANGPT and TEK. *p* values are indicated by color intensity. Note that significant interactions of ANGPT1 and TEK were only found in fibroblast/EC or SMC/EC pairs. *p* values are indicated by color intensity

Proper functioning of the ciliary body requires cooperative signaling arrangements of the epithelium, smooth muscle, immune cells and other stromal cells. We next focused our attention on ligand-receptor pairs among major ciliary cell types to infer potential intercellular communications in this tissue. Through CellPhoneDB (see Methods), we revealed that genes involved in a total of 1046 ligand-receptor pairs (1396 as the input repository) were present in our scRNAseq dataset. Based on the transcriptional expression levels of the ligand and receptor genes and the percentage of cells expressing these genes, we computed interaction scores of any possible ligand-receptor pairs between two cell types. After statistically analysis of the scores using a one-sided Wilcoxon rank-sum test, we identified 8,079 cell–cell interactions formed between homotypic or heterotypic cell pairs across the 14 human ciliary cell types (Fig. 6A). Fibroblast, PE, monocyte, NPE and EC were the top 5 cell types that contributed the greatest intercellular communication (Fig. 6A).

We then categorized the interactions into four subgroups based on the classification of the ligands, including ECM, chemokine, growth factor/hormone and membrane protein-mediated interactions (Fig. 6B). We found that growth factors/hormone contributed the most interactions in our scRNAseq data (Fig. 6B). Collagen family members represent the most common ECM ligands that interact with integrin receptors in human ciliary body (Suppl. Fig. 5A). Fibroblast, Schwann cell, SMC, NPE and PE were the major sources of collagen family members that strongly interact with the integrin receptors expressed in the cell clusters of Schwann cell, EC, SMC and fibroblast (Fig. 6C). As expected, myeloid cells including monocytes, macrophages, and neutrophils were majorly involved in chemokine/cytokine–receptor association (Suppl. Fig. 5B). Given the pathogenic roles of the ciliary body in uveitis, we wanted to highlight the intercellular communication that potentially contributes to intraocular inflammation. We selected the chemokine/chemokine receptor genes IL10 receptor (IL10R), IL15, IL15 receptor α (IL15RA) and the adhesion gene ICAM1 that were believed to be the common biomarkers of many forms of uveitis [44]. Surprisingly, our study failed to identify any interactions mediated by IL10R or IL15 in all ciliary cells. However, a variety of the highest-scoring interactions involved ICAM1 and IL15RA (Fig. 6D), indicating that they may be immune-mediated causes of uveitis.

Lastly, we found that fibroblast, PE, NPE and EC were the top 4 cell types involved in canonical growth factor-mediated cell–cell interactions (Suppl. Fig. 5C), and EGF, TGFβ, DLL4/Jagged, VEGF and FGF were among the most common growth factor family members that interact with their receptors in human ciliary body (Suppl. Fig. 5D). As revealed by the cell communication analysis, interactions between EGF family members and their receptors were majorly formed among NPE, PE, Schwann, EC, fibroblast and monocyte populations, suggesting EGF pathways may be commonly required for functioning of many ciliary cells (Fig. 6E). TGFβ and FGF were frequently coupled with their receptors within the fibroblast, NPE and PE populations (Fig. 6F, G), indicating highly activated TGFβ and FGF signaling in these cell types. DLL4/Jagged-Notch signaling was mostly activated between EC and PE clusters whereas EC and fibroblast served as the major target cells of VEGF ligands that were expressed by a variety of ciliary cells (Fig. 6H, I). Finally, as impaired ANGPT-TEK signaling is linked to risk of primary congenital glaucoma [45, 46], we examined ANGPT-TEK interaction in our scRNAseq dataset. We found that significant interactions of ANGPT1 and TEK were limited to fibroblast/EC and SMC/EC pairs, while interaction of TEK with the other major ANGPT ligands, ANGPT2 and 4 was not present in human ciliary body (Fig. 6J).

## Discussion

Cells that reside in the ciliary body play key roles in the ocular physiology as they constitute the anterior portion of the uveal tract and are responsible for the production of aqueous humor and vision accommodation. In this study, we generated a cell atlas for the ciliary body in humans using scRNAseq technology. Cluster analysis identified 14 distinct cell types, representing the epithelial, vascular, neural, stomal and immune cell populations of human ciliary body. We revealed cell-type discriminative gene markers or expression patterns and systemically inferred cell–cell interactions across the ciliary body tissue including those potentially involved in the pathogenesis of glaucoma and uveitis. Although a previous study has profiled the single-cell transcriptomics of ciliary cells in combination with the adjacent neural retina cells in the mouse eye [38], we provide the first comprehensive portrait of the cellular and molecular components and their interactions in human ciliary body.

In comparing the cellular constituents in human ciliary body with those in mouse ciliary body and the contiguous retina tissue [38], we found that most cell types, including the ciliary epithelium, SMC, vascular EC, fibroblast, melanocyte, Schwann cell, myeloid (macrophage and neutrophil) and lymphoid (T cell and B cell) cell populations, were present in both species, despite at varied frequencies. Commonly expressed molecular markers were mapped in the respective ciliary cells from both human and rodent (rat and mouse) eyes [29–33, 38–40, 47], indicating that the cell identities are generally conserved across these species. Other immune cell types such as monocytes, plasma and mast cells were identified in human ciliary body but were absent in the mouse datasets [38], possibly attributable to their comparatively low density in the ciliary body/retina. Interestingly, the presence of dendritic cells is well-documented within the entire uveal tract including the iris, ciliary body and choroid in a number of species [9, 10, 48–50], but others’ and our scRNAseq analyses failed to identify dendritic cell population in both human and mouse ciliary body with or without the mixture of other ocular tissues [16, 38, 47]. This could be due to the difficulties in completely dissociating/capturing the dendritic cells that reside between the firmly attached ciliary epithelium layers [9, 48, 49].

RAX is a homeobox-containing transcription factor highly expressed in retinal progenitor cells [51, 52]. RAX and other homeobox-containing transcription factors such as PAX6, SIX3 and LHX2 constitute a regulatory network to determine retinal progenitor cell fate, thereby promoting the formation of the neural retina and RPE during eye development [53]. In Xenopus, overexpression of *Rax* gene led to ectopic formation of the retina tissue and hyperproliferation of RPEs [51]. As NPE and PE are structurally continuous and developmentally related to the neural retina and RPE [54], it is unsurprising that RAX and the other homeobox genes are also specifically expressed in these ciliary epithelial cells but not in other ciliary cells without a neuro-epithelium origin. The essential roles of PAX6 [55] and LHX2 [56] in the appearance, differentiation, and morphogenesis of the ciliary epithelium layers have been documented previously, although little is known about RAX, SIX3 and many other potential marker genes in ciliary epithelial cells. Future studies validating the precise localization and functionalities of these genes in the ciliary epithelium layers are warranted.

Despite the common progenitor cells during early eye development, PE and NPE undergo distinct differentiation steps at the late embryonic stage [2] and eventually possess differential gene expression patterns. The human and mouse [38] datasets consistently showed that members of the classical ZIC family transcription factors ZIC1 and ZIC2 are upregulated in NPE compared to PE. ZIC proteins critically regulate tissue morphogenic signaling pathways such as Wnt, sonic hedgehog and nodal during embryonic development, particularly in neural tissues [57, 58]. The expression of ZIC proteins around mouse ciliary marginal zone has been documented previously [59]. Whether they specifically regulate the structural folding of NPE remains to be elucidated. With respect to specialized functionalities, the ciliary epithelium is considered the production hub for major components of zonule fibers and the vitreous humor [30] and our data indeed showed that NPE might be preferentially responsible for the production of these ECM components. Despite these differences, PE and NPE have very similar expression pattern of water/ion channel genes and tight junction components required for the production of aqueous humor and the assembly of blood-humor barrier. Although the precise working model of aqueous humor secretion has not been described, these findings provide clues for cells in the ciliary epithelium bilayer acting as a cooperative arrangement of water/ion transport and tissue barrier.

The secreted aqueous humor can be drained through both the conventional outflow pathway consisting of the trabecular meshwork and Schlemm’s cancel and the unconventional uveoscleral pathway involving interstitial spaces of the ciliary muscle [60]. The absence of classic draining lymphatics in normal ciliary body reinforces the notion that unconventional outflow may not involve the action of draining lymphatics within the uvea, i.e. the so called uveolymphatic outflow [13]. Although quantum dots or other tracers injected into the anterior chamber were shown to accumulate in cervical lymph nodes draining the ocular surface [13, 61, 62], they were not necessarily drained by lymphatic vessels within the ciliary body or other intraocular tissues, as these tracers can follow the alymphatic unconventional outflow pathway and be cleared by the conjunctival lymphatics subsequently. Nevertheless, neo-lymphatic vessels can invade the ciliary body in the presence of malignant melanoma with extraocular extension [14, 15]. Whether lymphangiogenesis occurs in the ciliary body under other pathological conditions, such as uveitis, remains to be answered.

The ciliary muscle controls lens accommodation to focus on near objects and facilitates the draining of aqueous humor into Schlemm's canal, and has been used as the therapeutic target to treat cycloplegia [63] as well as glaucoma [64]. However, it should be noted that the ciliary body not only indeed contains the ciliary muscle but also vascular smooth muscle, both of which are made of smooth muscle fibers. Despite the high similarity of their transcriptomics, these two cell types possess certain characteristic gene expression patterns. One example is desmin, which is known to be expressed in CMC but not in VSMC [39, 40]. Moreover, the preferentially expressed gene sets responsible for energy consumption nicely correlates with the greater abundance of mitochondria in CMC [65]. Collectively, this provides the basis for future investigations of the ciliary muscle in pathological conditions and raises interesting possibilities that targeting the preferentially expressed genes in CMC over VSMC might result in more specific therapeutic approaches against refractive error development or glaucoma.

One of the major cell types identified was the macrophage population, supporting the notion that cells of the myeloid lineage primarily make up the immune repertoire of the healthy uveal tract [9]. The ciliary body has been recognized to have an immunosuppressive environment [9, 66], and the anti-inflammatory (M2) macrophages are important for the maintenance of such an immune status. Still, a substantial proportion of the resident macrophages in human ciliary body are otherwise polarized towards a pro-inflammatory phenotype, which may be required for phagocytosis and clearance of cellular debris in the tissue [41]. Moreover, microglia sitting in the retina and non-microglia macrophages in the ciliary body/iris, like their counterparts in the brain, have a slow turnover rate and were thought to be independently maintained via self-renewal [9, 67, 68]. This notion has been challenged by the identification of extra-retinal origin of repopulated retinal microglia from ciliary body/iris macrophages [43]. In agreement with this, we showed here the presence of a macrophage-like microglia subpopulation in human retina, which was likely derived from the ciliary M2 polarized macrophages. The differentially enriched pathways between M2 macrophage-like microglia and the other microglia, such as NFκB and NOD-like receptor pathways, are well-known contributors to microglia/macrophage differentiation and activation [69–71]. This predicts that signals involved in the control of ciliary M2 macrophage polarization might be important in constantly contributing to the resident microglia pool in the neuroretina. Despite these findings, the donors’ health conditions and other biometrics should be considered about when interpreting data from a human study. For example, the differential proportions of immune cell populations, especially macrophages and the relative ratio of M1/M2, in donor 3 vs. donors 1&2 may be attributed to his end stage lung cancer and/or elder age. Although the transplantation of the avascular corneas donated by most cancer patients (except those with certain blood or eye cancers) is considered safe [72], and donor patient 3 had no invasive cancer cells in the ciliary body as well as clinical signs of ocular metastases, the potential impact of malignant cancer (particularly lung cancer in this case) and/or the therapeutic interventions on triggering infiltration/activation of macrophages (while reciprocally reducing the proportion of other immune cell populations) in the ciliary body may be present and warrants further study. Moreover, how certain forms of intracranial hemorrhage (e.g., in donor 1 vs. 2) differentially affect local immune environment and cellular heterogenicity in the ciliary body also remains unclear. In terms of the effects of aging, previous studies have shown that macrophage accumulation and glial reactivity were elevated in the retina of older mice [73] and such responses may also be present in the ciliary body tissue of elder patients (e.g., in donor 3).

The CellPhoneDB computational approach allowed for the identification of potential cell–cell interactions in human ciliary body microenvironment. The demonstration of collagen as the most common ligand involved in ECM-integrin interactions, despite that the ciliary body is renowned as the origin of zonule fibers that are primarily made up of fibrillin [74], predicts the major role of the collagen matrix in the repetitive structure support and cell adhesion within the ciliary body tissue during accommodation, and the signaling events behind this type of interaction may also promote the differentiation and survival of nearby cells [75]. Of note, ECM components produced by the ciliary body are also required for the stabilization and turnover of the inner limiting membrane and the vitreous body as their structural proteins, such as laminin and collagen, originate primarily from the ciliary body and the lens [6]. Therefore, a deeper understanding of the mechanistic roles of collagen deposition and signaling network is of significance. A growing body of studies have verified that the morphogenesis of the ciliary body is dependent on classic growth factor pathways such as TGFβ, FGF, Notch, WNT/β-catenin and Hedgehog [2, 3, 76], and the identification of varying numbers of cell–cell interactions mediated by differential growth factors and their signaling receptors may be quantitatively related to their functionalities in the patterning, cell type specification and differentiation of ciliary cells. In particular, the observations that TGFβ, FGF and DLL4/Jagged are intensively involved in NPE and PE-mediated intercellular communication in the ciliary body are in line with their critical roles as local paracrine signaling molecules driving ciliary body morphogenesis [2, 3]. EGF signaling may have broad roles in the proper functioning of the ciliary body whereas VEGF signaling specifically targets vascular endothelial cells and fibroblasts as well. Finally, of further interest is the identification of cell–cell interactions involving ANGPT1-TEK, ICAM1 and IL15RA which were previously attributed to glaucoma [45, 46] and uveitis [44], respectively. Yet, despite the lack of patient ciliary body tissue collection in the present study, building a communication network of the pathogenic factors and their source cells may help guide the understanding of the etiologies of these diseases and the development of useful therapies just as the inhibition of PD1-PDL1-mediated interaction has been proven to be effective in cancer therapy [77, 78].

In summary, our study has revealed the molecular taxonomy that controls the epithelium and smooth muscle functionality, the transitional states of resident macrophage and the cellular communication in human ciliary body at single-cell resolution, thus bringing new understanding to the regulation of intraocular fluid and immune homeostasis under physiological and pathological conditions.

### Limitations of the study

When working with scRNAseq samples from postmortem human tissues, a significant limitation is the exceeding lability of the RNA molecules. Although a previous study has documented that the death-to-preservation time lag (2–6 h) indeed resulted in lower RNA integrity of the ciliary body tissue, longer postmortem time (8–48 h, when compared to < 6 h) did not appear to further impair the RNA integrity in human ciliary body [79]. In our study, the time elapsed from the donor patients’ death until the generation of live single cell suspension was between ~ 5 and 10.5 h and the overall time lag from death until the completion of cDNA libraries was between ~ 8 and 13 h. We believe that this remains within acceptable limits of scRNAseq sample preparation duration and that our data are representative of the physiological human ciliary body status albeit influences from the time lag could not be avoided from an ethical point of view. Moreover, the varying fractions of cell types (especially immune cells) among the three donor samples may be attributed to their distinct health/aging conditions; yet, this requires further in-depth investigation using a large-scale cohort of human samples and experimental disease models. Lastly, the enriched pathways and cell–cell communication inferred here remain to be validated by future functional studies.

## Supplementary Information

Below is the link to the electronic supplementary material.

Supplementary file1 (PDF 4774 KB)

Supplementary file2 (XLSX 12 KB)

Supplementary file3 (XLSX 12 KB)

Supplementary file4 (XLSX 50 KB)

Supplementary file5 (XLSX 22 KB)

Supplementary file6 (XLSX 27 KB)

Supplementary file7 (XLSX 15 KB)

Supplementary file8 (XLSX 11 KB)

## Data Availability

The raw and unprocessed sequencing data files generated in this study are available in NCBI GEO database (accession #GSE206026) and CNCB-NGDC database (accession #HRA003051). All reported data are included in the manuscript and in the source data files.
