# Supplementary material for: A single-cell transcriptomic atlas of the human ciliary body
Source: Cell Mol Life Sci. 2022 Sep 26;79(10):528. doi: 10.1007/s00018-022-04559-w (PMC9512889; doi:10.1007/s00018-022-04559-w)
Supplement: Supplementary file 1 — Supplementary file1 (PDF 4774 KB) [file 18_2022_4559_MOESM1_ESM.pdf]

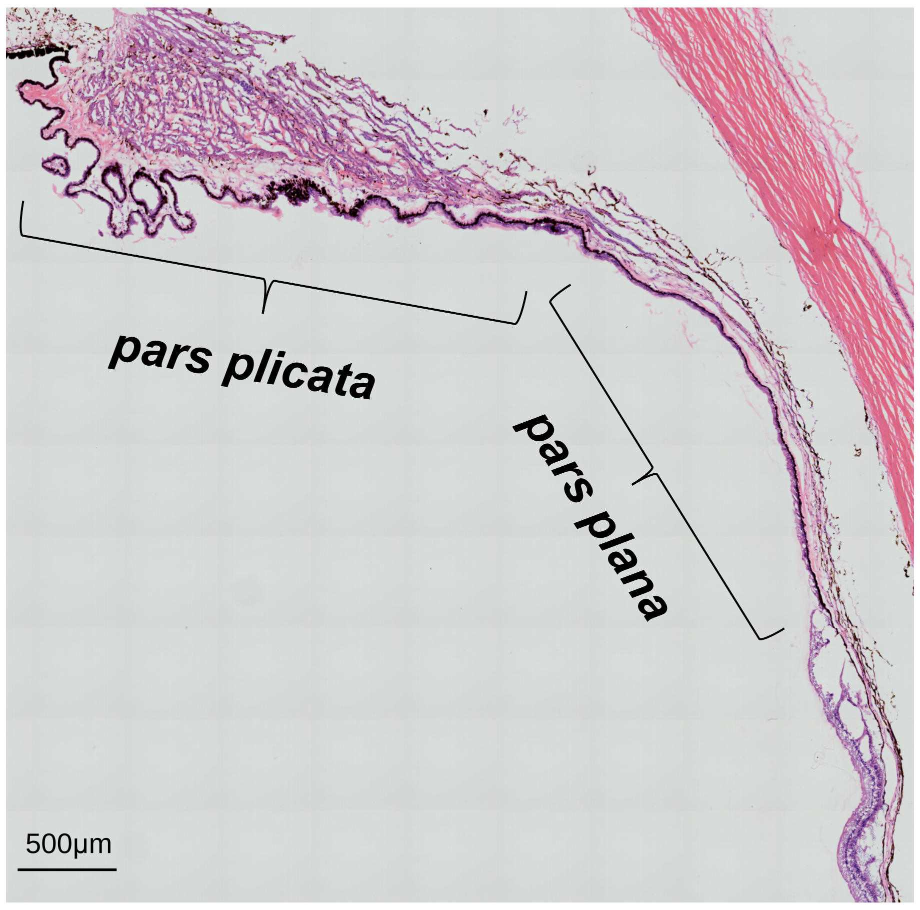

Suppl. Figure 1 Histology of human ciliary body. Shown are the *pars plicata* and *pars plana* regions of the ciliary body and contiguous tissues in the cross-sections of the human eye with Hematoxylin-Eosin (HE) staining.

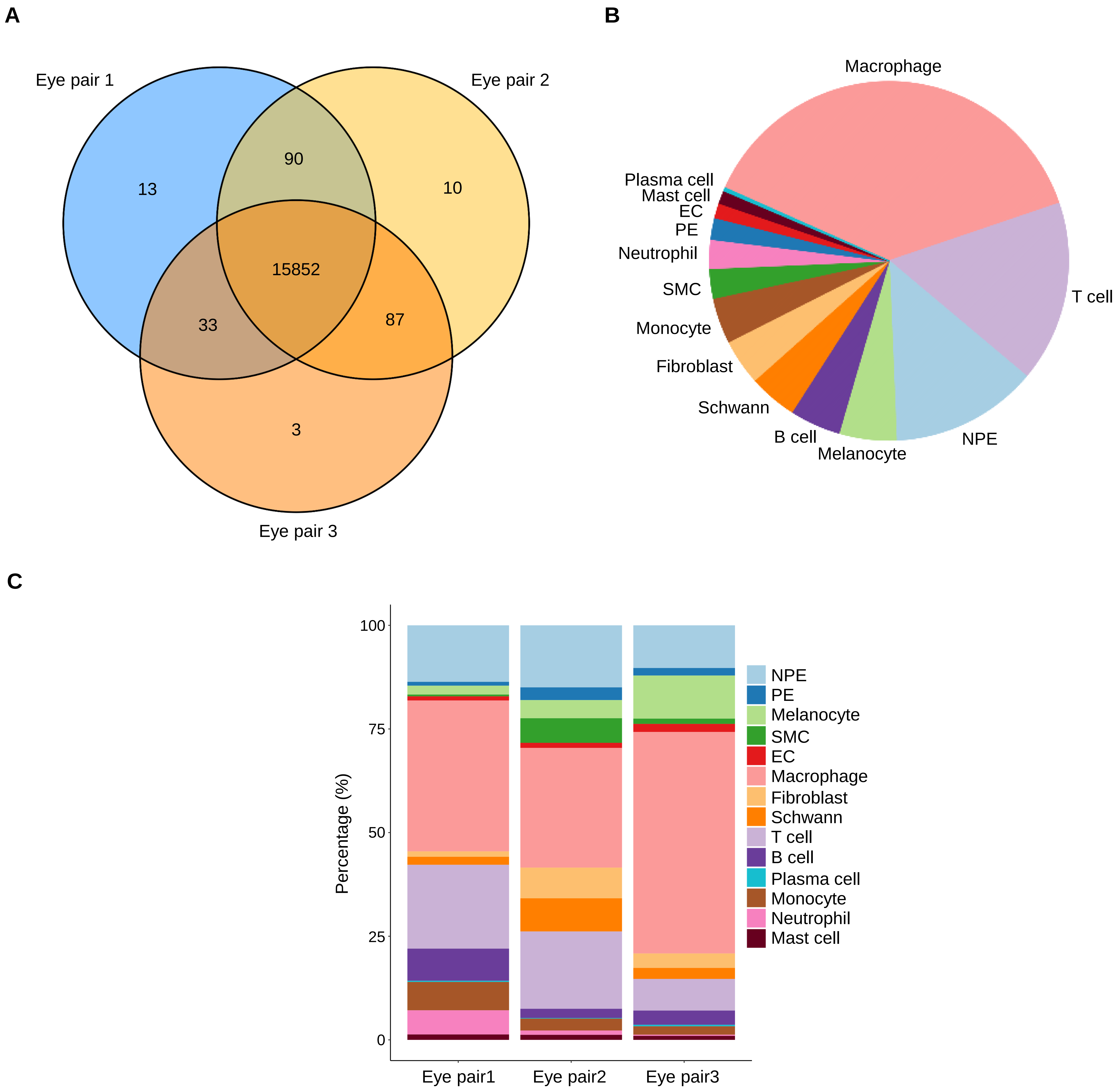

Suppl. Figure 2 (related to Figure 1)

(A) Venn diagram comparing the genes detected in ciliary cells from 3 pairs of human donor eyes. Note that 15,852 out of the total 16.088 genes were detected in all three samples.

(B) Proportion of the 14 ciliary cell types identified in scRNAseq from the 3 eye pair samples.

(C) Percentage of ciliary cell types in each eye pair sample.

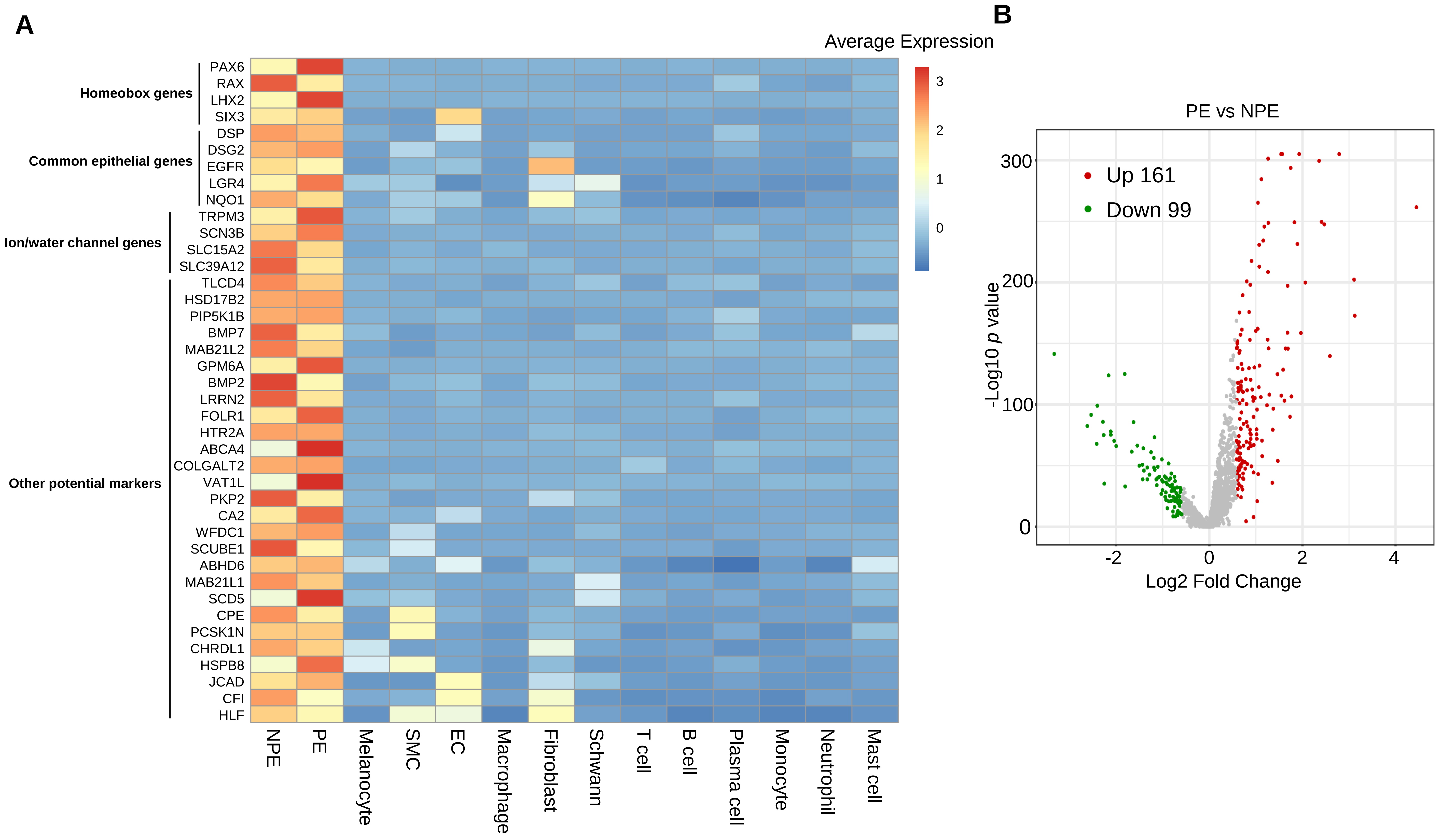

Suppl. Figure 3 (related to Figure 2)

(A) Heatmap showing averaged expression levels of the identified potential marker genes specifically expressed in both PE and NPE. Expression levels of the genes are labeled by color intensity. A ranked list of these genes is shown in Suppl. Table 4. Color intensities indicate average expression.

(B) Volcano plot analysis of differentially expressed genes (DEGs) in PE vs NPE of human ciliary body. Upregulated and downregulated genes are labelled in red and green, respectively.

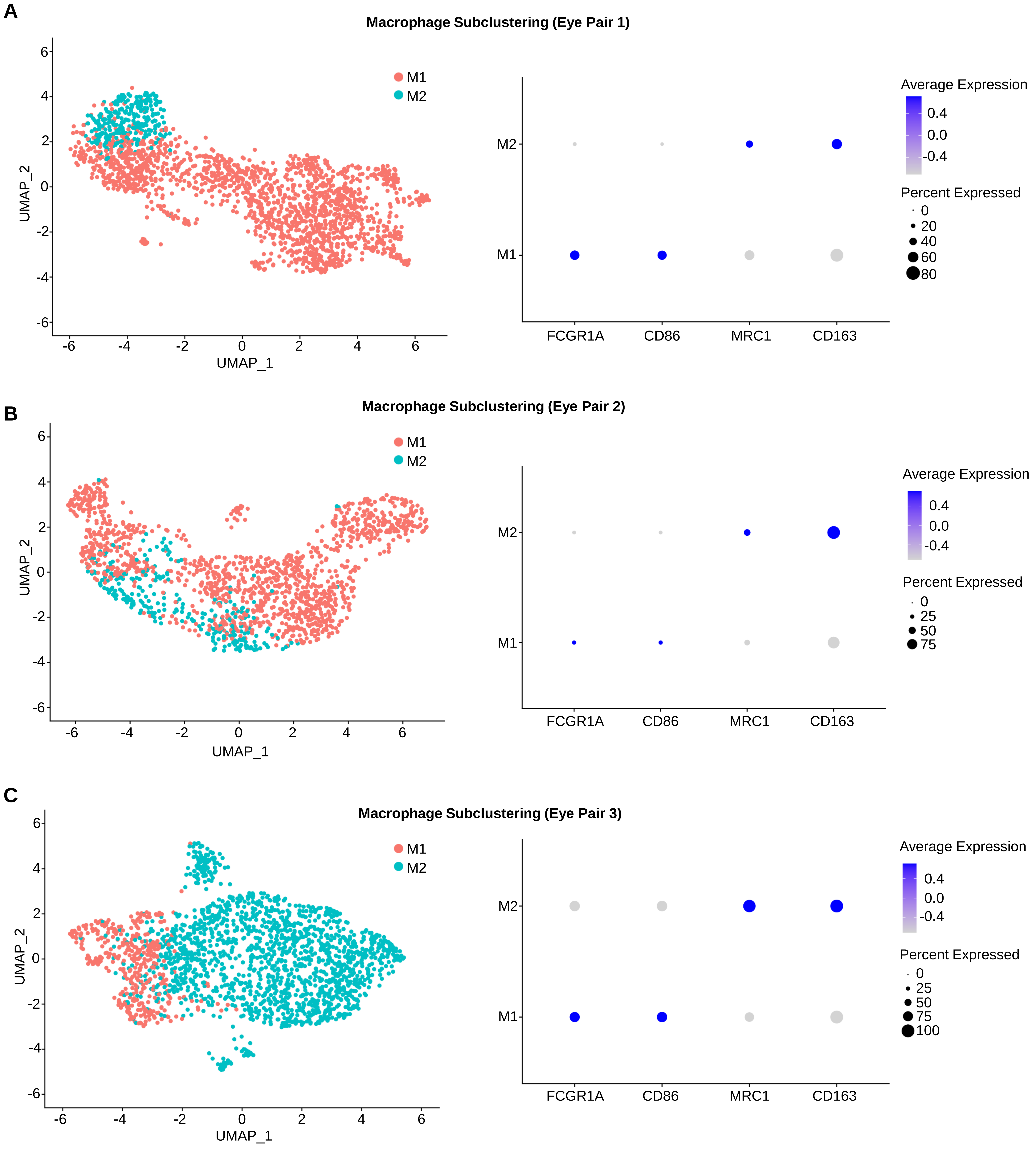

Suppl. Figure 4 (related to Figure 5)

(A-C) UMAP visualization and dot plot of expression levels and frequency of selected markers of M1- and M2-polarized ciliary macrophages in each donor eye pair sample.

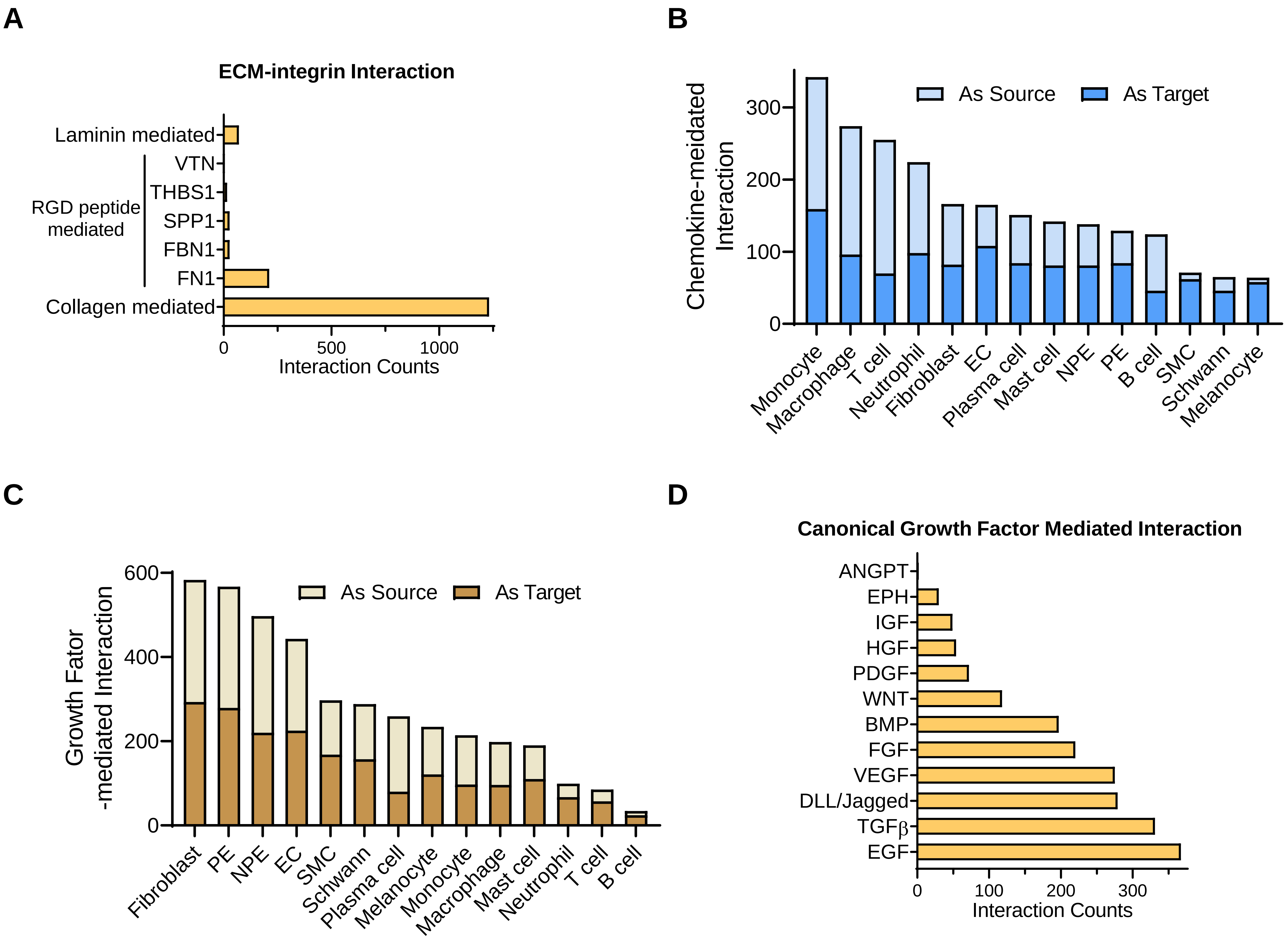

Suppl. Figure 5 (related to Figure 6)

(A) Quantifications of ECM-integrin interactions across all human ciliary cell types. Shown are counts of significant ciliary cell-cell interactions mediated by collagen, RGD-containing protein and laminin superfamily members and their integrin receptors.

(B) Quantifications of chemokine-chemokine receptor interactions across all human ciliary cell types. Shown are counts of significant interactions among human ciliary cell types that act as the source of chemokines and the target (receptor) of the interaction pairs.

(C) Quantifications of growth factor-mediated interactions across all human ciliary cell types. Shown are counts of significant interactions among human ciliary cell types that act as the source of growth factors or the target (receptor) of the interaction pairs.

(D) Quantifications of canonical growth factor-mediated interactions across all human ciliary cell types. Shown are counts of significant cell-cell interactions mediated by EGF, TGFβ, DLL/Jagged, VEGF, FGF, BMP, WNT, PDGF, HGF, IGF, EPH and ANGPT superfamily members and their receptors.
